# Multiple calcifying hyperplastic dental follicles: a major diagnostic consideration in multiple pericoronal lesions - report of two cases

**DOI:** 10.1186/s12903-020-01146-y

**Published:** 2020-06-01

**Authors:** Israel Guardado-Luevanos, Anna Jazmine Haro, Marisol Godínez-Rubí, Jorge Alejandro Puente-de los Santos, Jaime Aguirre-Macías, Diana Paloma Soltero-Chávez, Miguel Padilla-Rosas, Mario Nava-Villalba

**Affiliations:** 1 Master’s Program in Oral Pathology and Medicine, Department of Integral Dental Clinics, University Centre of Health Sciences, Universidad de Guadalajara, Sierra Mojada No. 950, Col. Independencia, C.P. 44340 Guadalajara, Jalisco Mexico; 2 Laboratory of Pathology Research, Department of Microbiology and Pathology, University Centre of Health Sciences, Universidad de Guadalajara, Sierra Mojada No. 950, Col. Independencia, C.P. 44340 Guadalajara, Jalisco Mexico; 3 Maxillofacial Surgery Service, Hospital General de Zapopan O.P.D., Ramón Corona 500, Col. Centro, C.P. 45100 Zapopan, Jalisco Mexico; 4 Inova Dental Clinic, Mar Marmara 2001, Col. Country Club, C.P. 44610 Guadalajara, Jalisco Mexico; 5 Nucleo Dental, Dental Clinic, Av. López Mateos 567, Col. Ladrón de Guevara, C.P. 44600 Guadalajara, Jalisco Mexico

**Keywords:** Pericoronal radiolucencies, Pericoronal lesions, Multiple jaw radiolucencies

## Abstract

**Background:**

Pericoronal radiolucent lesions are a common radiographic finding, but it is rare that they occur in multiple forms. Multiple calcifying hyperplastic dental follicles (MCHDF) are entities with few cases described to date; nevertheless, they appear to have a very particular phenotypic pattern.

**Cases presentation:**

Case 1: A 10-year-old male was evaluated radiographically, revealing four impacted canines, each accompanied by unilocular pericoronal radiolucency. Case 2: A 16-year-old male was planning orthodontic treatment; following his radiological evaluation all third molars were found to be accompanied with pericoronal radiolucencies. Enucleation, and third molar removal along with the pericoronal tissue were the respective treatments. Microscopically, in both cases, the specimens shown odontogenic epithelium, and type I and II calcifications in the hyperplastic follicles, all these characteristics were consistent with MCHDF.

**Conclusion:**

Although MCHDF are a rare entity, they must be considered in the differential diagnosis of multiple pericoronal lesions. Under the light of the current evidence, the histological findings may be relatively heterogeneous, but their integration with both the clinical data, which are apparently particular, and with the radiographic characteristics, can lead to a definitive diagnosis.

## Background

Pericoronal radiolucent lesions may have different origins, and the possibilities represent a wide range of entities (Table 1). In general, the lesions mentioned in Table 1 are commonly identified as radiographic findings. However, they are usually solitary and unilateral, and observing these types of lesions in either multiple and/or bilateral form, except for odontogenic keratocysts and lesions associated with genetic disorders, is rare [1–4]. Pericoronal radiolucencies are often detected when patients undergo radiologic evaluation as a result of unerupted teeth, and in many cases the proposed diagnostic probability is “multiple dentigerous cysts” [5, 6]. In practice, this possibility is unlikely, since cases of multiple dentigerous cysts are particularly infrequent [1, 2, 4], in addition, as Table 1 refers, there is a wide variety of lesions that, similarly to dentigerous cysts, could present as pericoronal lesions. In this sense, although multiple calcifying hyperplastic dental follicles (MCHDF) are rare, they have a consistent phenotypic pattern. We herein report two new cases of MCHDF and discuss their clinical-radio-pathological features which support identifying MCHDF as a major initial diagnostic consideration in multiple pericoronal lesions.

**Table 1:** Groups of lesions with pericoronal

Aetiology	Entity
Developmental conditions	Hyperplastic dental follicle**** Single calcifying hyperplastic dental follicle* Multiple calcifying hyperplastic dental follicles* Multiple hyperplastic dental follicles*
Odontogenic cysts	Dentigerous cyst**** Eruption cyst *** Odontogenic keratocyst*** Orthokeratinized odontogenic cyst** Calcifying odontogenic cyst**
Odontogenic tumours	Unicystic ameloblastoma** Solid/multicystic ameloblastoma** Ameloblastic fibroma** Adenomatoid odontogenic tumour** Odontoma (premineralized stage)* Calcifying epithelial odontogenic tumour* Odontogenic myxoma* Central odontogenic fibroma* Ameloblastic fibro-odontoma*^,a^ Squamous odontogemic tumour* Primordial odontogenic tumour* Archegonous cystic odontoma*^,b^
Other neoplasms	Langerhans cell disease* Ossifying central fibroma*^,c^
Malignant neoplasms	Intraosseous mucoepidermoide carcinoma* Carcinoma arising in dentigerous cyst* Ewing’s sarcoma*
Genetic disorders^d^	Gardner Syndrome* Cleidocranial Dysplasia* Gorlin-Goltz Syndrome* Noonan Syndrome* Mucopolysacaridosis* Pyknodisostosis*
Rarities	Hemophilic pseudotumour*

****, ***, **, *Relative visual scale for frequency of occurrence; ^a^Their neoplastic or hamartomatous nature is still under debate; ^b^Controversial entity with primordial odontogenic cyst; ^c^The 2017 WHO classification includes it in the category of benign mesenchymal odontogenic tumors. ^d^Radiolucencies that accompany the impacted teeth

## Cases presentation

### Case 1

**Fig. 1:**
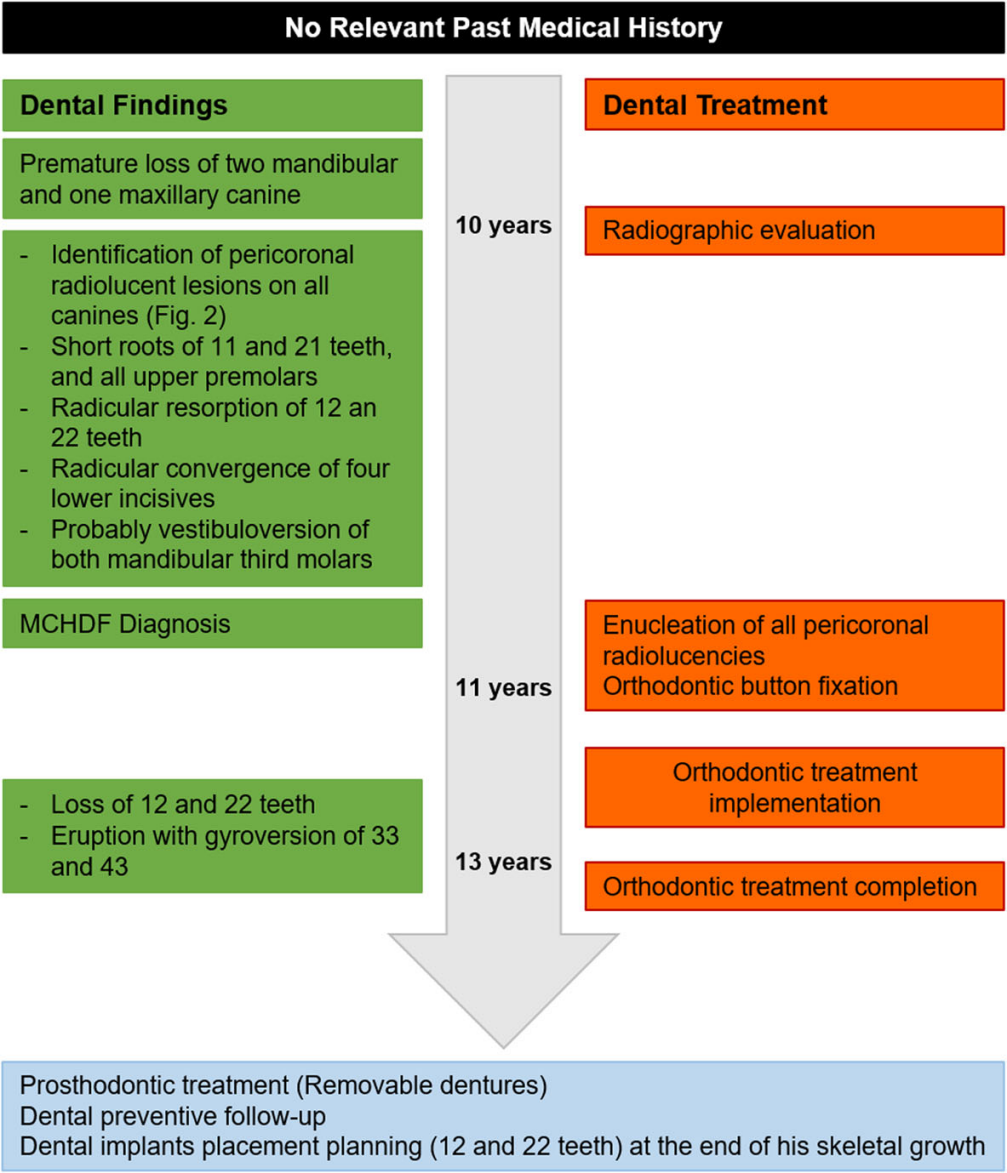
Timeline case 1

**Fig. 2:**
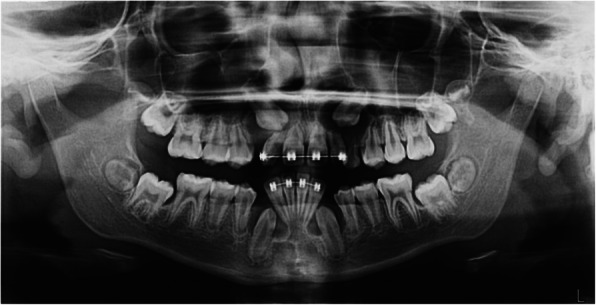
Panoramic radiograph showing pericoronal radiolucencies affecting the four canines. Distal inclination of mandibular incisors and radicular resorption of the maxillary lateral incisors is also present. Several teeth show apparently short roots

A 10-year-old male with premature loss of the two primary mandibular canines and the primary right maxillary canine, was evaluated in a dental clinic (Fig. 1). During the examination, a slight dental malposition of the anterior segments was also noted. The medical history was not contributory. A panoramic radiograph showed four impacted canines, each accompanied by a pericoronal radiolucent area of approximately 4 mm widened. (Fig. 2). Short roots of the maxillary centrals and a root resorption process on the lateral incisors were also observed, and a presumptive diagnosis of “multiple dentigerous cysts” was made. The lesions were enucleated using local anaesthesia and an osteotomy was performed through the alveolar crest with manual instrumentation. Finally, an orthodontic button was placed on exposed crowns.

**Fig. 3:**
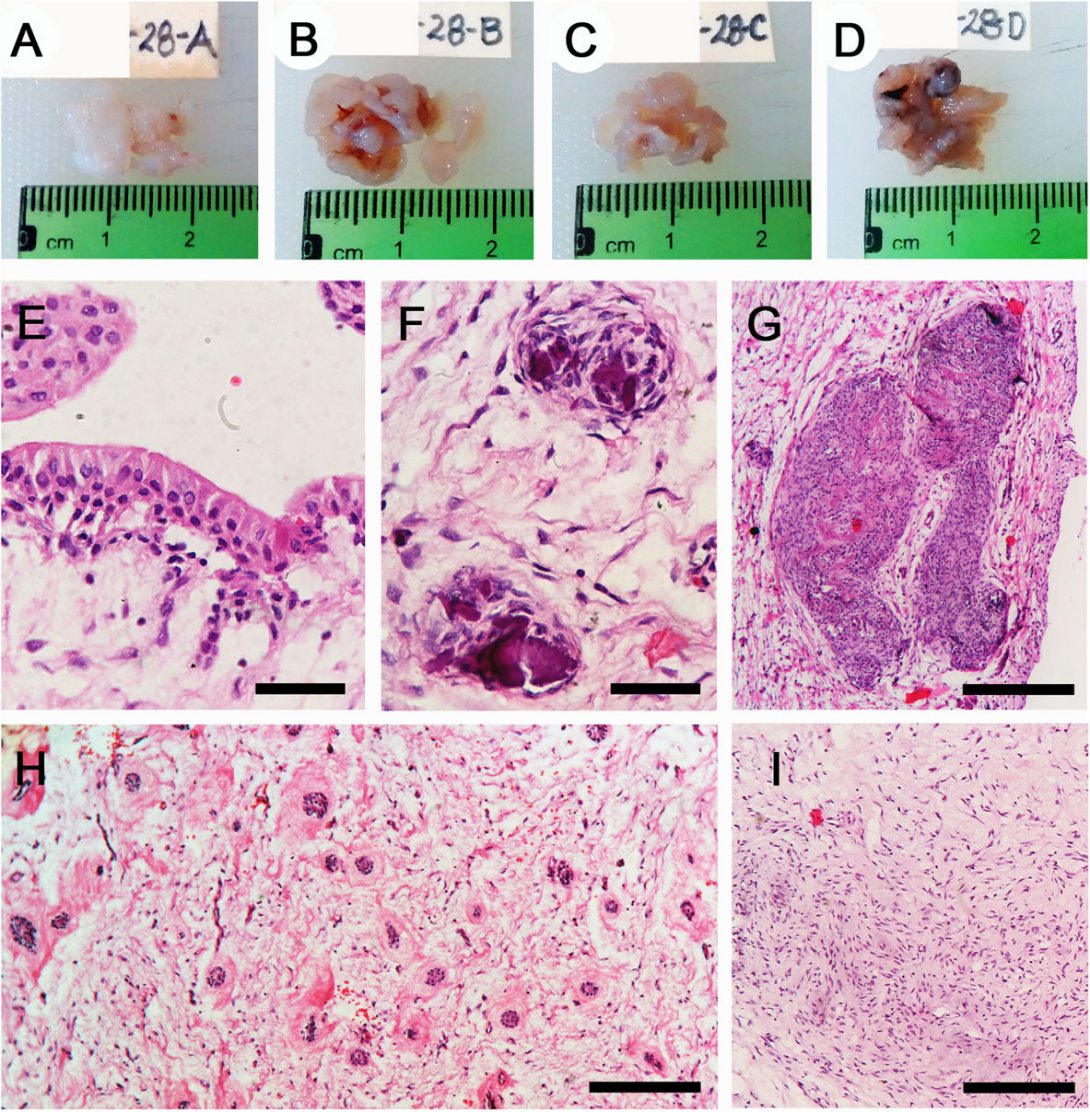
Macroscopic and histological findings of follicles, case 1. **a-d** Saccular conformation of the specimens, in the order corresponding to teeth 1.3, 2.3, 3.3, 4.3 (*FDI* notation), white to brownish coloration and dimensions larger than one centimetre can be noted. **e** Reduced enamel epithelium found in different luminal sections. **f** Type I calcification observed in all follicles in a scattered pattern. **g** Type II calcification observed in the follicles of 1.3 and 4.3 teeth. **h** Multiple islands of odontogenic epithelium with peripheral hyalinization. **i** Foci of mesenchymal condensation in the follicles thickness of the 1.2, 2.3, 4.3 teeth (photomicrographs stained with haematoxylin and eosin, original magnification to E and F × 40, scale bar 50 μm; original magnification to G-I × 10, scale bar 200 μm)

**Fig. 4:**
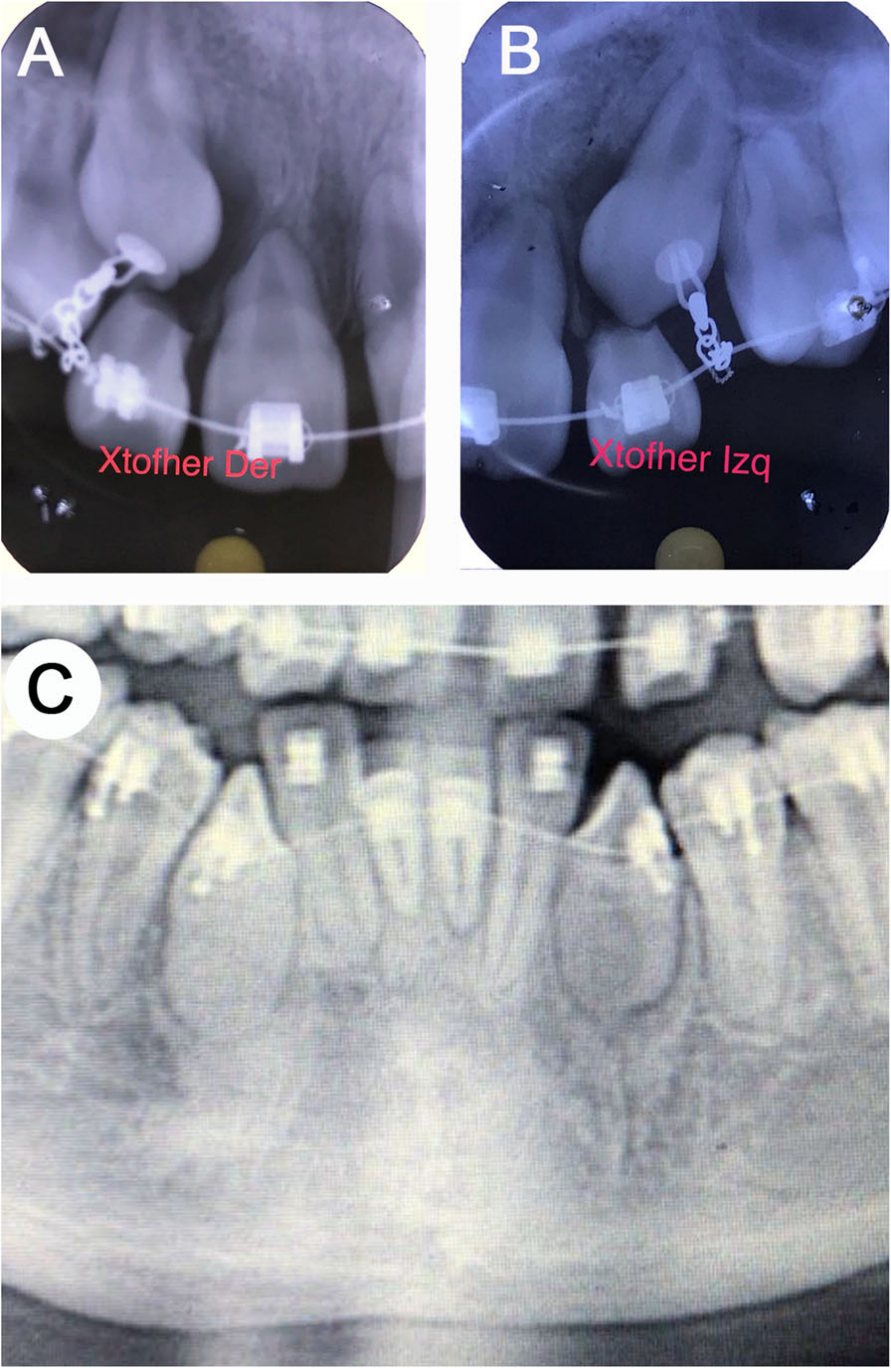
**a-b** Radiographic monitoring showing the orthodontic traction process and the root resorption progression of the maxillary lateral incisors (**a**: 1.3, **b**: 2.3 teeth periapical radiographs). (**c**) Anterior section of the panoramic radiograph showing the eruption of the mandibular canines with gyroversion, the distal inclination of the mandibular incisors has decreased

Macroscopically, the four specimens had a saccular aspect of a fibrous, resilient, white tissue (Figs. 3a-d). Microscopically, the four specimens had heterogeneous histopathological features (Table 2), but in general were constituted by hyperplastic follicles with loose fibrous connective tissue, which on its luminal surfaces were partially covered by reduced enamel epithelium (Fig. 3e). The presence of type I calcification was consistent in all specimens, but scant and disperse (Fig. 3f). Type II calcification was focal, and present only in 1.3 and 4.3 specimens (*FDI* notation) (Fig. 3g). In all follicles the presence of odontogenic epithelial islands could be noted, several of them showing peripheral hyalinisation (Fig. 3h). Finally, focal zones of mesenchymal condensation were seen in at least 3 specimens (Fig. 3i). The clinical and radiopathological correlation was consistent with MCHDF. Unfortunately, there was no active participation during the orthodontic treatment and this caused the collapse of the maxillary canine dental spaces, and the progression of radicular resorption to the cervical region of the maxillary laterals (Fig. 4). After 2 years, all canines had formed roots and the lower canines had erupted; they did, however, present gyroversion and short roots.

**Table 2:** Microscopic characteristics of cases

Histopathological Feature	Case 1	Case 2
Reduced enamel epithelium	1.3, 2.3, 3.3, 4.3	1.8, 2.8, 3.8, 4.8
Odontogenic epithelial rests	1.3, 2.3, 3.3, 4.3	1.8, 2.8, 3.8, 4.8
Type I calcification	1.3, 2.3, 3.3, 4.3	1.8, 2.8, 3.8, 4.8
Type II calcification	1.3, 4.3	1.8, 4.8
Follicle with predominantly dense fibrous connective tissue	–	3.8, 4.8
Follicle with predominantly loose fibrous connective tissue	1.3, 2.3, 3.3, 4.3	1.8, 2.8
Mesenchymal tissue condensation	1.3, 2.3, 4.3	3.8

*FDI* notation is used to identify affected teeth

### Case 2

**Fig. 5:**
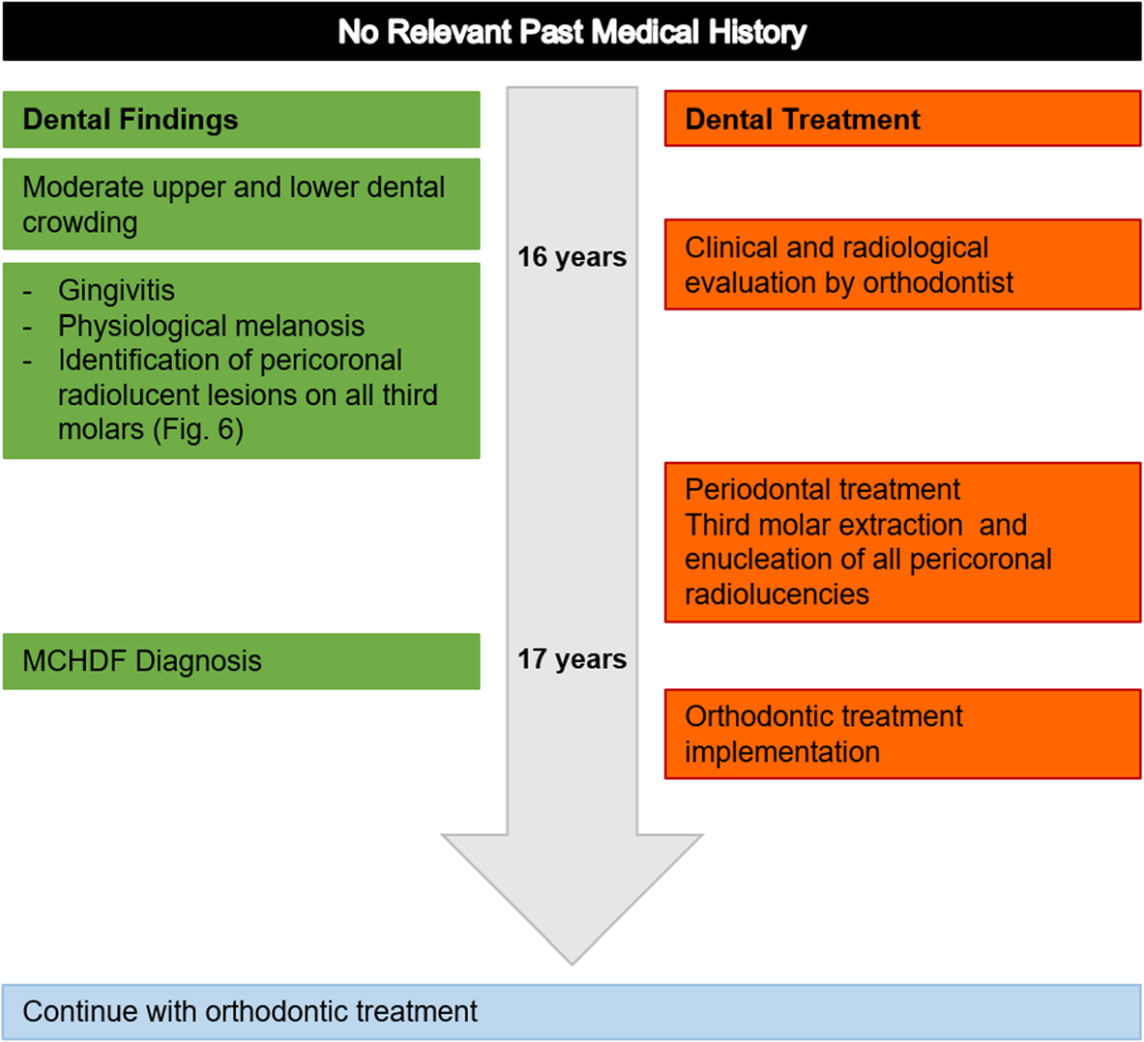
Timeline case 2

**Fig. 6:**
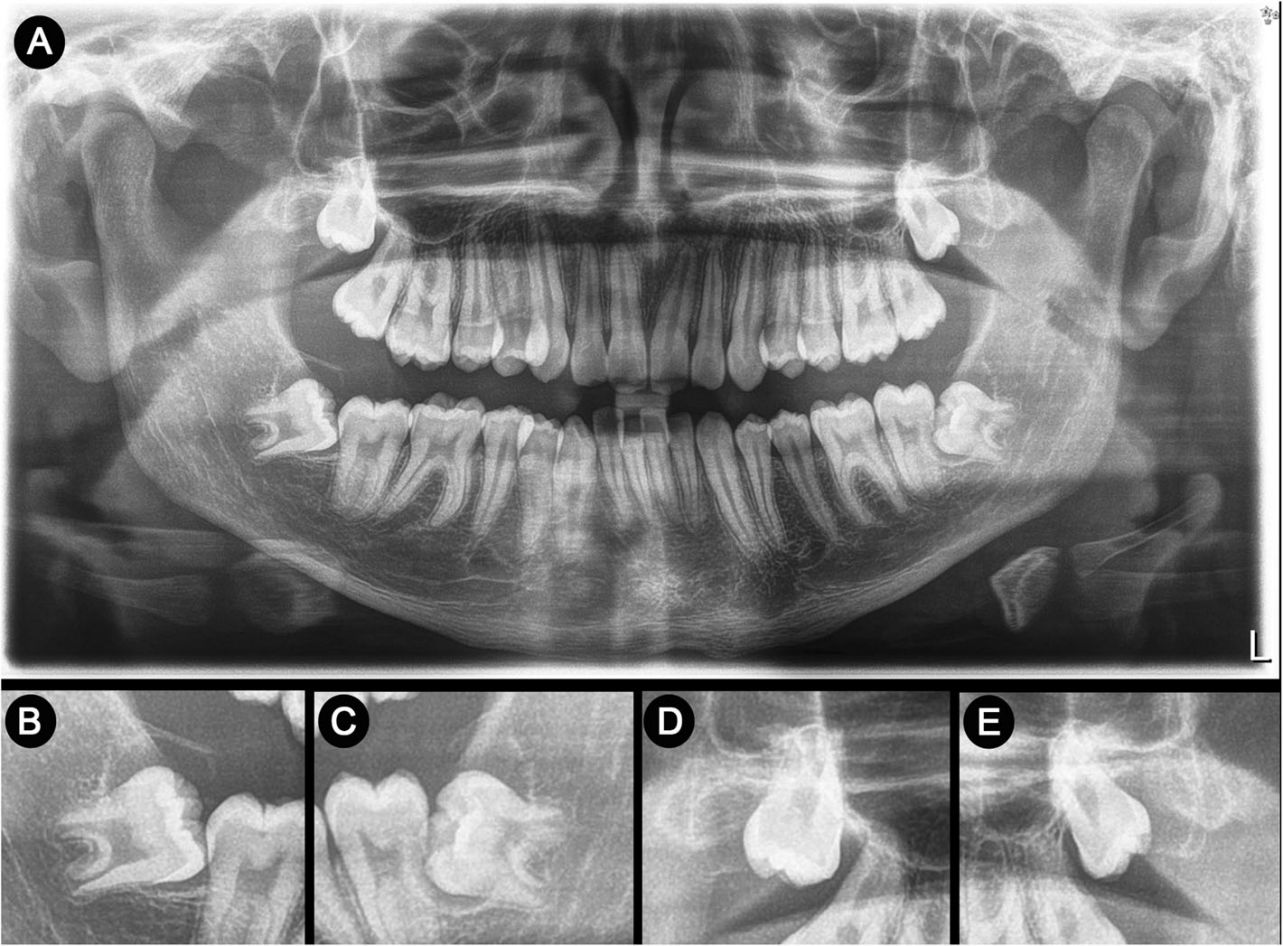
**a** Panoramic radiograph showing pericoronal radiolucencies affecting the four third molars, radiopaque borders can be identified. **b**-**c** Closer images of the mandibular third molars, showing a wide radiolucent pericoronal areas, delimited by thin radiopaque borders. **d**-**e** The maxillary third molars show pericoronal radiolucent areas with radiopaque borders that originates from the cervical margins, which eventually presents a vanishing towards the alveolar crest, however, during the surgical procedure, pericoronary tissue was prominent and easily identified

A 16-year-old male went to a private clinic to plan his orthodontic treatment (Fig. 5). During the intraoral examination, slight crowding, mild gingivitis, physiological melanosis and some carious lesions were observed. The past medical history was not relevant for the oral condition. In the radiographic evaluation, large radiolucent areas of 4–5 mm widened, were observed surrounding the four third molars (Fig. 6). The extraction of these teeth was scheduled once his dental rehabilitation and periodontal treatment were completed (he turned 17 during this time), the presumptive diagnosis of radiolucencies was “multiple dentigerous cysts”, and the surgical approach was uneventful.

**Fig. 7:**
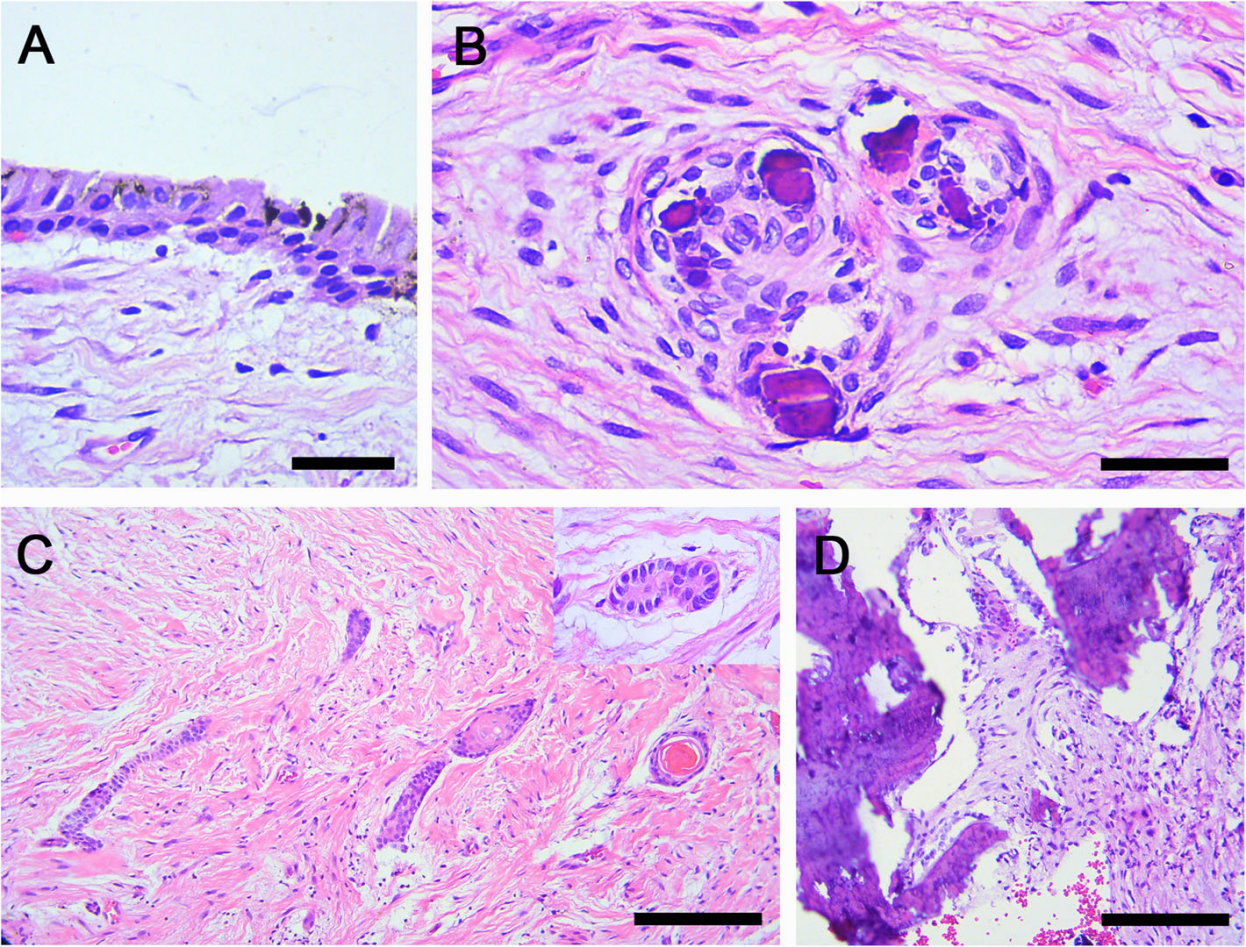
Histological findings of follicles, case 2. **a** Reduced enamel epithelium occasionally present in luminal surface. **b** Calcification type I scattered identified in all follicles. **c** Cords and islands of odontogenic epithelium with squamous metaplasia. **d** Calcification type II observed in the follicles of 1.8 and 4.8 teeth (*FDI* notation) (haematoxylin and eosin, original magnification to A-B and inset C × 40, scale bar 50 μm; original magnification to C-D × 10, scale bar 200 μm)

The macroscopic appearance evidenced saccular structures, with a light brown coloration, smooth surface and resilient consistency. Heterogeneous histopathological findings were appreciated in the different follicles (Table 2). The connective tissue varied between dense and loose, and the luminal surface was occasionally covered by reduced enamel epithelium (Fig. 7a). Type I calcifications were present, but scant and disperse (Fig. 7b). Throughout the thickness of the follicles, islands of odontogenic epithelium could be observed, in varying amounts with respect to each follicle and in some islands there was even squamous metaplasia (Fig. 7c). Type II calcifications were only present in the follicles of the 1.8 and 4.8 teeth (*FDI* notation) (Fig. 7d). Areas of condensation of mesenchymal tissue were scarce. A final diagnosis of MCHDF was established. After 8 months of follow-up, the healing areas have evolved optimally and the patient is concluding his orthodontic treatment.

## Discussion and conclusion

**Fig. 8:**
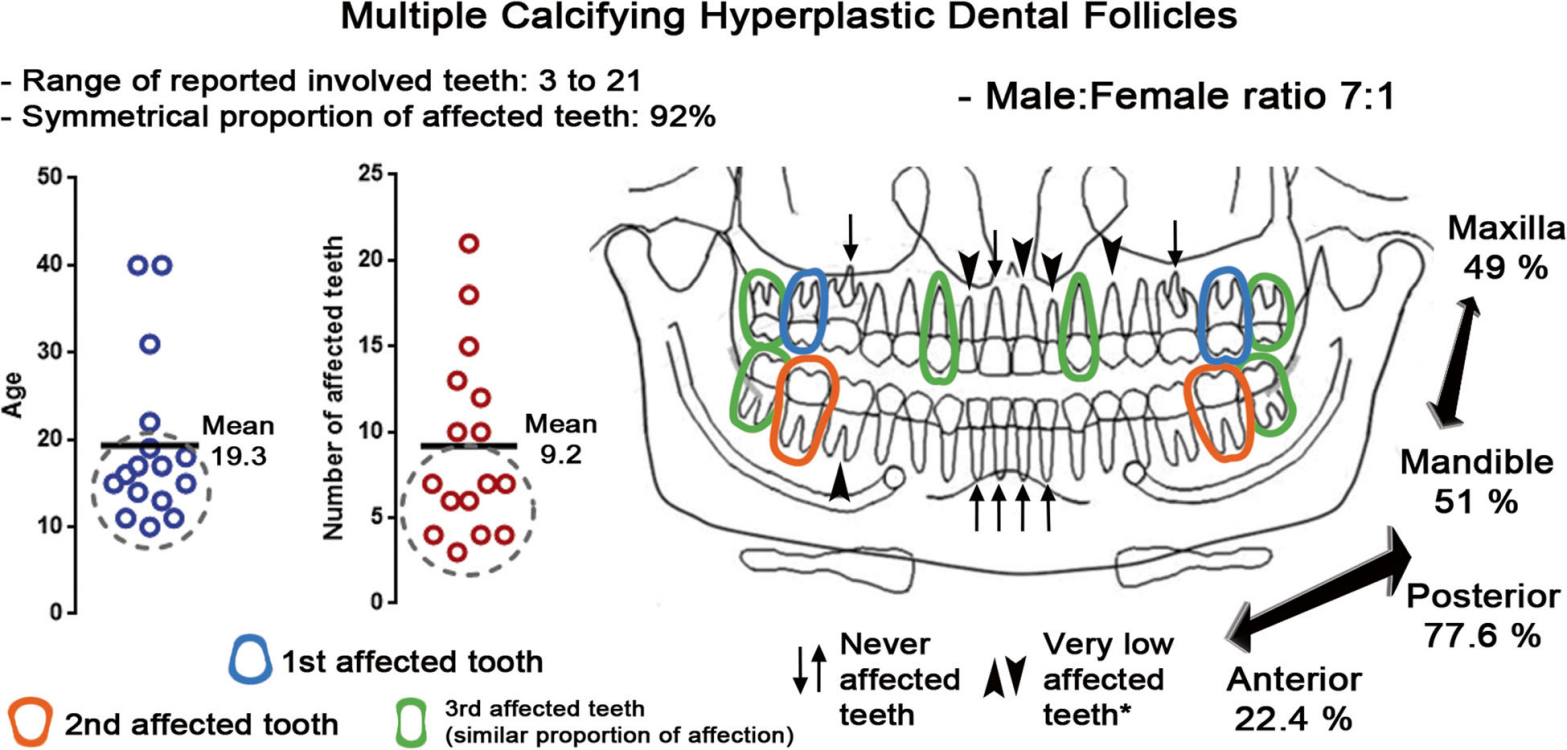
Summary of demographic characteristics, as well as the number and teeth affected by MCHDF, (asterisk) teeth involved only once

To date, there have been few cases of MCHDF reported, in this discussion we have considered those that cover the following parameters: three or more teeth involved and with no association with dental or syndromic abnormalities [5–14]. A graphic summary of the information obtained from the 16 cases under consideration, can be seen in Fig. 8. In this image, we can see that MCHDF predominantly affects pubertal/adolescent men, with between 3 and 9 teeth usually affected, especially in the posterior region, and with both the maxilla and mandible affected in the same proportion. Only 3 cases (19%) reported to date are of patients between 30 and 40 years old. Although MCHDF can affect the teeth of the maxillary anterior segment, canines are by far the most affected. The mandibular incisors, as well as the first maxillary molars, have never been reported to be affected by MCHDF. On the other hand, if we compare the affected teeth bilaterally, we can recognise that there is a 92% symmetrical pattern, a particularity that has not been previously mentioned in the literature. Delving into the peculiarities of this entity highlights the fact that the second molars are the teeth most affected (Fig. 8), a situation that is quite rare and had already been mentioned in previous studies [6, 15]. Whereas the affection pattern of teeth seems to be a distinctive feature, the involvement of some cases or teeth is up for debate. For example, some authors do not consider third molars to be among the teeth affected by MCHDF [6, 13], presumably due to the early age at which they were identified or diagnosed, and because these teeth tend to impact. Moreover, the adjacent soft tissue of third molars could also have calcified material and odontogenic epithelium [16, 17]. We believe, however, that there are several parameters which support considering the third molars to be teeth affected by MCHDF: 1) their affectedness is in the context of multiple involvement, 2) the most affected region are the posterior quadrants, 3) they exhibit enlarged pericoronal radiolucencies, 4) adult patient cases display third molar involvement [11–13], 5) and as in our case, these teeth have bilateral symmetric involvement. With respect to the last point, there is a case report covering histological features of calcifying hyperplastic dental follicles, but only two teeth are affected, and it has a unilateral presentation; interestingly, the lesions are in proximity [8]. It is difficult, however, to establish at present whether it is MCHDF or contiguous single calcifying hyperplastic dental follicles. MCHDF can affect the deciduous [13] and supernumerary teeth [10] and can be associated with dental agenesis [9] or the presence of paramolar teeth [6]. They can cause cortical expansion in both the maxilla and mandible [6, 11, 13]. MCHDF can also be associated with congenital phenotypic changes, not necessarily a syndromic established pattern [6, 7] or systemic alterations (epilepsy, mental retardation, hematuria/hypercalciuria or hypothyroidism) [6, 11], without knowing if there is a direct relationship. So far, whether there is a vertical family component has not been established, but two brothers affected by MCDHF have been reported [6]. Given the fact that MCHDF are associated with multiple impacted teeth, it is reasonable to consider various syndromes accompanied by this phenomenon, among them Gardner Syndrome, Cleidocranial Dysplasia, Gorlin-Goltz Syndrome, Noonan Syndrome, Mucopolysacaridosis, and Pyknodisostosis [18, 19], although each of these has important phenotypic features which rule them out. It is nonetheless important to consider those syndromes in which the phenotypic characteristics are accentuated with development, as in the case of Gorlin-Goltz Syndrome, which usually presents multiple odontogenic keratocysts as early manifestations, which in turn can be seen as wide pericoronal radiolucent lesions [20], or Gardner Syndrome, in which multiple impacted teeth are a common and premature finding, but whose intestinal manifestations are a considerable risk [21]. These syndromes should therefore be considered in differential diagnosis work when there are cases of multiple pericoronal lesions.

From the radiographic point of view, MCHDFs are usually identified due to the lack of teeth eruption. In this sense, although the common trait of MCHDF is calcification, this is not radiographically evident in the early stages and is therefore insubstantial, but has been demonstrated to be very premature [6, 9]. In patients with a long (∼33.2 year old, range 22–40 year old) [8, 11–13] or progressive evolution [9, 11], it is possible to observe radiopacities or increase them within their pericoronal radiolucencies, and the use of periapical radiographs is recommended to demonstrate this fact [11]. Several authors have reported sclerotic borders as a substantial radiographical finding [9–12, 14]. Some approaches have been made to determine abnormalities in dental follicles [22], where the proposed limit width measure is 2.5 mm in periapical radiography and 3 mm on the panoramic radiograph [11, 23]; above these ranges the dentigerous cyst is a possibility. However, in the case of MCHDF, this parameter may not be useful, given the few cases described to date, and because the report of this measure is variable, with some cases reporting an amplitude of 6 mm [8], others 11 mm [10], and most cases not registering the measure at all. There also seems to be a relationship between time progression and the extent of the follicle [8, 11, 12]. On the other hand, although MCHDF are hamartomatous lesions, they can induce radicular resorption like our present Case 1; although the authors do not mention it, in the case reported by Davari [13] there seems to be root resorption in right maxillary first molar. It has also been radiographically possible to identify the fact that MCHDF are able to displace the inferior alveolar canal [13] or cause dental tilt as in Case 1.

Diverse approaches have been made in their treatment, the most common being follicle removal, together with the associated dental organ. The single enucleation of the hyperplastic tissue has a variable success rate in inducing affected tooth eruption, and it is better to accompany it with orthodontic traction [6, 12]. During the surgical approach, the pericoronal tissue’s firm adherence to the bone has been noted [7, 9, 12], and varying degrees of bone cortical thickness can be present [7]. The absence of a cystic cavity is striking [7, 9]. Marsupialization has been attempted without success [6] and long-term follow-up is suggested for untreated cases [11]. Despite appearing to be an obvious consequence, we believe it is important to mention that those teeth affected by MCHDF will develop short roots, and thus their orthodontic or prosthetic use may be compromised. An excellent review of the approach and management of cases of delayed dental eruption is available [19].

With respect to pathological features, macroscopically, a grating sound during sectioning [5, 7, 9, 14] and nodulations in the specimen surface [7, 9, 14] are the most relevant characteristics reported, the first feature was consistent in our cases. After making an evaluation of the available literature, it should be noted that the histopathological findings of the different follicles are usually not homogeneous (Table 2) [6, 13]. In relation to odontogenic epithelium, some cases have peripheral hyalinisation around odontogenic epithelial islands [5, 7]; in others the epithelium undergoes squamous metaplasia [6] or has clear cell changes [6, 9, 13, 14]. Peripheral hyalinisation (Fig. 3h) and squamous metaplasia (Fig. 7c) was detected in our cases 1 and 2, respectively. Additionally, in all follicles in our cases, reduced enamel epithelium was present, at least partially (Figs. 3e, 7a and Table 2), but this is not mentioned in all cases of MCHDF.

In the cases of young patients (≤11 year old), there is a loose fibrous connective tissue which seems to become denser as age progresses [6, 13]. In both our cases, the establishment of the hyperplastic process was relatively short based on the dental eruption chronology, and we thus observed loose or at least mixed dense-loose connective tissue (Figs. 3h, 7c and Table 2). Another feature that we observed and that has not been reported were zones of condensation of mesenchyme-like tissue (Fig. 3i); we do not interpret these as “induction phenomenon” since there were no associated odontogenic epithelial islands, although we believe that it is likely to be a very incipient arrangement of type II calcification.

As for calcifications, in some cases a certain type predominates [6, 8, 11, 12, 14]; the calcification can be florid [8, 12, 13], in groups [9] or scarce and dispersed [6]. In the cases presented here, type I calcification was predominant but scarce. Moreover, some MCHDF do not present type II calcification [5–7, 14]. Likewise, there are apparently diverse calcification patterns; in the case of type I calcification there are seemingly two arrangements, which are probably associated with time progression, and apparently its nature is non-collagenous [24]. The earliest calcification pattern shows acellular, small, basophilic and irregular structures, immersed into fibroblasts and collagen fibers forming a whorled pattern [6, 14]; the second calcification pattern shows spherical bodies, with concentric lamellar apposition similar to Liesengang’s rings, immersed in the whorled pattern, but generally not as cellular [6, 9–12]. Both cases presented here show the first type I calcification pattern described.

Type II calcification has at least three arrangements: 1) a swirling spindle cell pattern intermixed with mineral deposit areas presenting tufting on borders, and well-defined interfacing with surrounding stroma [6]; 2) a wider multiple spherular calcification pattern and more diffuse edges, similarly to focal type I calcifications but expanding and colliding [5, 7, 9–11, 13], and 3) those cases in which calcification is cellular, irregular, mineralized and trabecular, surrounded by a discrete condensation of the stromal tissue [6, 8, 11]. We were able to identify the first type II calcification pattern in case 1 presented here (Fig. 3g), and we also identified the third type II calcification pattern in case 2 (Fig. 7d).

Type II calcification is fibrillar in nature, as demonstrated by polarized light [8, 12] and can trap type I concentric calcifications [8] (a good example of this phenomenon of entrapment of type I calcifications inside type II calcifications is shown in this case [25] [we are not considering MCHDF here]). Additional histological findings associated with MCHDF, such as pulpal calcifications, have also been described [9]. Thus, while MCHDF have distinctive histological features, they are usually of variable proportions and must be complemented by radiological and clinical integration.

It has already been proposed that the possible origin of calcifications could come from mesenchymal cells, which can differentiate into cementoblasts or osteoblasts [13, 26], and odontogenic epithelium may play an inducing role [7, 9, 26] although the lack of digestion of the fibrous tissue is the strongest thesis for impacted teeth on HDF [26]. Approaches in this regard have proposed a failure in the remodeling microenvironment with the downregulation of some metalloproteases in HDF [27], but the calcification induction remains unresolved. In line with these observations, in the case of lesions that have similar calcification patterns, it has been suggested that failed eruption could be the inducer of hamartomatous or cystic changes in pericoronal dental follicular tissues [28]. A fine detail about the characteristics of the patients affected by MCHDF is that neither the first molars (at least the maxillary ones) nor the mandibular incisors are affected, indicating that whatever the process, activation or deregulation, it is triggered at around 5 years of age according to the chronological formation of the crowns. Nevertheless, it could be even more premature as in the case of a primary right maxillary second molar being affected [13]. Another important association of the disease is its predominance in males, giving rise to the possibility of hormonal influence [13, 14], although this possibility is questionable since two cases have recently been reported affecting women [11, 12]. Interestingly, these cases are both adult women (31 and 40 years old), with a 100% symmetrical dental condition pattern, and of Turkish origin. Further studies are necessary to determine what the precise cause or aetiology of this entity might be.

Attempts have already been made on several occasions to clarify the difference between Calcifying or not, Hyperplastic Dental Follicles (HDF) with Simple or WHO type Central Odontogenic Fibroma (COF). The succinct difference in the COFs, in addition to the cementoid calcifications and the odontogenic epithelium, is the greater cellularity, greater vascularity and the occasional presence of dentinoid or osteoid material [8, 29, 30]. In fact, based on the polarization colours of Picrosirius red-stain, a significant difference may be found in the COF thick fibrillar component (1.6–2.0 μm), which were significantly more green and greenish-yellow as compared with those from HDF [31], although in MCHDF it seems that these results are not always applicable [5, 12]. Some authors have proposed Congo red stain (negative result) to rule out calcifying epithelial odontogenic tumours [7, 13]. However, this discussion seems already to have been surpassed, as the WHO currently considers pericoronal COF-like lesions as HDFs [32]. In this sense, there is already enough evidence showing that the calcification patterns identified in MCHDF and originally described in Regional Odontodysplasia [33] occur in a variety of syndromic conditions [25, 34] or in association with dental abnormalities [24, 35–38]. Therefore, we only consider in our summary (Fig. 8) cases of “pure” MCHDF; all other associations may be part of a much broader spectrum of follicle-dental involvement, their approach exceeds the objectives of this text and should be considered in a possible review article.

The study of MCHDF is not without controversy. A few years ago, an interesting and provocative critique of an article was published, in which it was suggested that MCHDF may have descriptions dating back from 1945 [39]. Critics also drew attention, to the diagnostic pitfalls in cases of multiple lesions, if clinical and radiopathological integration is not performed, and gave two case reports as examples [40, 41]. The criticized articles are interesting reports of multiple radiolucent pericoronal lesions, and a good example of atypical clinical presentation of multiple odontogenic tumours. Finally, another case described in Japan is suggestive of MCHDF in a 14-year-old male, with the 4 s molars affected, clinically interpreted as “dentigerous cysts”. Histopathological findings show hyperplastic dental follicles, but unfortunately there is no reference to whether calcifications were found [26]. These controversies only demonstrate the rich diversity of manifestations in which the alterations of the organism are expressed, and the ways in which we interpret them.

In conclusion, a definitive diagnosis must be supported by clinical-radio-pathological correlation because there exist similar lesions such as multiple hyperplastic dental follicles (not calcifying) [26, 42] or single calcifying hyperplastic dental follicles (not multiple) [6, 26, 43]. As previously mentioned, several conditions, principally tumours and cystic lesions, can present pericoronal location (Table 1). However, the occurrence of multiple forms is rare [1–4, 40, 41], hence MCHDF should be considered instead of multiple tumours or cystic lesions, as other authors have already mentioned [11]. MCHDF should therefore be suspected in male patients between 10 and 20 years old with 3 or more impacted teeth, without syndromic phenotype, involving maxillary/mandibular second molars and/or maxillary/mandibular canines, and accompanied by enlarged pericoronal radiolucencies with symmetrical patterns and without the involvement of the inferior incisors and maxillary first molars. Histopathological examination, together with the clinical and radiological correlation, will determine the definitive diagnosis.

## Data Availability

All data generated or analysed during this study are included in this published article.

## Abbreviations

COF: Central odontogenic fibroma
FDI: *Fédération Dentaire Internationale*
HDF: Hyperplastic dental follicles
MCHDF: Multiple calcifying hyperplastic dental follicles
WHO: World Health Organization
